# Advanced Glycation End-Products in Blood Serum—Novel Ischemic Stroke Risk Factors? Implication for Diabetic Patients

**DOI:** 10.3390/jcm13020443

**Published:** 2024-01-13

**Authors:** Aleksandra Kuzan, Anna Kozak-Sykała, Anna Fiedorowicz, Wojciech Kałas, Leon Strządała, Andrzej Gamian

**Affiliations:** 1 Department of Biochemistry and Immunochemistry, Wroclaw Medical University, 50-367 Wroclaw, Poland; 2 Neurology and Stroke Department, Independent Public Healthcare Centre, Jankowski Regional Hospital in Przeworsk, Szpitalna 16, 37-200 Przeworsk, Poland; annak@spzoz-przeworsk.pl; 3 Hirszfeld Institute of Immunology and Experimental Therapy, Polish Academy of Sciences, 53-114 Wroclaw, Polandwojciech.kalas@hirszfeld.pl (W.K.); leon.strzadala@hirszfeld.pl (L.S.); andrzej.gamian@hirszfeld.pl (A.G.)

**Keywords:** advanced glycation end-products, ischemic stroke, transient brain attack, diabetes, vascular complications, cerebrovascular diseases

## Abstract

New predictors of ischemic incidents are constantly sought since they raise the awareness of patients and their doctors of stroke occurrence. The goal was to verify whether Advanced Glycation End Products (AGEs), in particular AGE10, could be one of them. The AGE10 measurement was conducted using a non-commercial ELISA assay in the blood serum of neurological patients without cerebrovascular event (n = 24), those with transient brain attack (TIA) (n = 17), and severe ischemic stroke (n = 35). Twice as many of the people with TIA or severe stroke presented high AGE10 serum concentrations compared to the patients with other neurological conditions (χ^2^ = 8.2, *p* = 0.004; χ^2^ = 8.0, *p* = 0.005, respectively). The risk of ischemic incident was significantly risen in people with higher levels of AGE10 (OR = 6.5, CI95%: 1.7–24.8; OR = 4.7, CI95%: 1.5–14.5 for TIA and stroke subjects, respectively). We observed a positive correlation (r = 0.40) between high AGE10 levels and diabetes. Moreover, all the diabetic patients that had a high AGE10 content experienced either a severe ischemic stroke or TIA. The patients with high levels of AGE10 exhibited higher grades of disability assessed by the NIHSS scale (r = 0.35). AGE10 can be considered a new biomarker of ischemic stroke risk. Patients with diabetes presenting high AGE10 levels are particularly prone to the occurrence of cerebrovascular incidents.

## 1. Introduction

Ischemic stroke is caused by blood artery occlusion by either locally forming a thrombus or embolus. A full-blown ischemic stroke often causes permanent neurological damage. In contrast, a minor stroke, namely a transient brain attack (TIA), is a short ischemic episode which does not evoke permanent deficits in patients, and its symptoms usually disappear within 1 h of its onset [1]. There are several conditions which particularly predispose to the occurrence of ischemic incidence. However, common stroke risk factors, such as hypertension, hyperlipidemia, or diabetes [2], often do not provoke patients to improve their self-surveillance for stroke. Moreover, even TIA, which increases the probability of severe stroke incidents in the near future dramatically [3], is frequently downplayed by patients, and they do not perform medical examinations when the symptoms are gone. Thus, new risk factors, which could indicate the increased probability of ischemic events stronger than the common stroke-related diseases or show the augmented risk of stroke in certain pro-ischemic conditions, are constantly sought since they would raise the awareness of people at risk and their doctors.

Among the risk factors, diabetes is particularly noteworthy. It is associated with chronic hyperglycemia, and glucose and other reducing sugars are involved in numerous mechanisms related to inflammation, atherogenesis, and vascular impairment, leading to macro- and microangiopathy and other clinical complications [4], of which strokes are particularly dangerous because they can have lethal effects. Among other things, glucose is a substrate in glycation and the higher the level of hyperglycemia, the more intense this process may be. Our concept was to associate one of the products of glycation and brain cerebrovascular events and analyze this relationship.

The Advanced Glycation Products (AGEs) are derived from the condensation of the carbonyl groups of reducing sugars or low-molecular-mass α-oxoaldehydes with free amino groups of proteins, or amino acids, where the first step is the formation of a reversible Schiff base [5,6,7]. This unstable bond undergoes an Amadori rearrangement, creating a more stable Amadori compound, considered an early product of glycation, a precursor of further compounds formed in the process of glycation. Subsequent reactions involving the complex cascade of dehydration, fragmentation, condensation, oxidation, and cyclization reactions yield a variety of advanced Maillard reaction end-products, so-called AGEs. In the presence of oxygen, the Amadori products are degraded to reactive dicarbonyl compounds; therefore, the term glycoxidation is also used for these reactions [7,8]. There are known only a few AGE structures, which comprise pentosidine, pyrraline, carboxymethyllysine (CML), derivatives of imidasole, argpyrimidine, cypentodine, arginine-lysine imidazole, imidazolone, or glycolaldehyde-pyridine (GA-pyridine) [5,9,10,11,12]. The AGEs are heterogeneous in mixtures, with a characteristic fluorescence [11,12]. A free endogenous and exogenous food-derived AGE may bind to its specific receptors RAGE and can stimulate cellular response [11]. The AGE–RAGE interactions contribute to and are responsible for the induction of oxidative stress, expression of adhesion molecules, and activation of apoptosis processes [13,14,15]. The AGE levels are relevant in the development of several diseases [16,17,18,19], but are particularly important in diabetes [7,20,21]. They are formed and accumulated in tissues [22,23]. Such products are also associated with aging processes [24,25,26]. 

Recently, we have reported on the synthetic melibiose-derived glycation product MAGE, which mimics a unique epitope present in human and animal tissues [27]. This MAGE product was synthesized in anhydrous conditions, different to the classic synthesis in water solution. The physiological serum epitope called AGE10 was determined with ELISA using an anti-MAGE monoclonal antibody. The AGE10 epitope is formed in physiological conditions most likely in other biosynthetic pathways than those known. The MAGE cross-reactive auto-antibodies were detected in patients with diabetes [27] and the epitope itself was also analyzed in the serum of patients with various types of metabolic diseases [28,29].

The aim of this study was to establish whether the levels of AGE10 in the blood serum are altered in patients with acute ischemic stroke and TIA in comparison with neurological patients without cerebrovascular event and whether AGE10 content can be considered a novel risk factor for cerebral ischemic incidence. 

## 2. Materials and Methods

### 2.1. Patient Characteristic and Blood Collection

The general characteristics of the patients are presented in Table 1. The 76 participants were enrolled from Neurology and Stroke Department of the Hospital in Przeworsk, wherein 35 patients were diagnosed with severe ischemic stroke, 17 with TIA, whereas 24 patients were admitted with non-stroke neurological conditions, such as epilepsy, different types of neuropathies or sciatic neuritis and were considered as controls. All control patients were AGE10-matched with those with cerebrovascular event. Subjects with ischemic stroke were diagnosed symptomatically and based on CT and MR scans. TIA patients were identified as presenting symptoms specific to stroke, which have resolved within 24 h from the onset and did not cause permanent neurological decline. The brain scans of participants with TIA did not show ischemic-like changes. The neurological deficits in ischemic patients were evaluated at admission using the NIH Stroke Scale (NIHSS). Subjects who suffered from cancer or those with a history of cancer were excluded from the study. The common biochemical examinations were conducted in all patients in the ischemic and control groups. The cut-off values for hypertriglyceridemia and hypercholesterolemia were set up as 150 mg/dL for triglyceride and 140 mg/dL for total cholesterol levels, respectively. Patients with diabetes were identified either on the basis of medical history and glucose tolerance test or were newly diagnosed by assessing concentrations of fasting glucose and hemoglobin A1c in the blood. Renal disease was recognized by measuring the levels and clearance of creatinine and estimated glomerular filtration rate (eGFR).

In summary, the admission criteria were stroke or non-stroke neurological conditions, diagnosed in detail using imaging tests. Exclusion criteria were current or past cancer and age below 40 years.

Blood samples were collected between 2 and 8 days after TIA, and 2 and 9 days from stroke occurrence. After drawing, blood was immediately used for serum separation. The serum was kept at −30 °C until use. The blood collection procedures were performed according to the Declaration of Helsinki and approved by the local ethics board—The Regional Medical Chamber in Cracow, Poland (OIL/KBL/11/2017). All participants signed an informed consent form to participate in the study.

### 2.2. Determination of AGE10 with ELISA

The test was performed essentially according to a slightly modified published procedure [28,30]. For the determination of AGE10, samples of serum (50 μL) were pretreated with proteinase K from Tritirachium album (Sigma Aldrich, Saint Louis, MI, USA) with 30 U/mg activity (concentration of proteinase K in each serum was 0.3 mg/mL) at 55 °C overnight in Eppendorf tubes under parafilm to release AGE10, prior to their use as inhibitors in the test. Increasing concentrations of the synthetic MAGE standard, corresponding to the serum AGE10, was used to give a calibration curve in the range of 0–1.6 μg. The MAGE product was obtained by high-temperature microwave synthesis and isolated by liquid chromatography (column with HW-55S gel in 0.01 M ammonium acetate buffer) [30]. Ninety-six-well MaxiSorp (Nunc^®^, Darmstadt, Germany) plates were covered with synthetic high-molecular-mass AGE (HMW-AGE) based on myoglobin, obtained by high-temperature microwave synthesis. The coating process lasted 4 h at a temperature of 37 °C. In the next step, the plates were blocked with 10% skim milk for 18 h at 4 °C. Monoclonal non-commercial anti-MAGE antibodies, produced at the Institute of Immunology and Experimental Therapy in Wrocław, in culture supernatant, were diluted with TBS at 1:100 dilution. Additionally, a control test in which there was no reaction with primary antibodies was performed. Mixtures of samples or LMW-AGE and antibodies were applied to the plate and after 2.5 h of incubation at room temperature, the plate was washed and treated with a solution of secondary antibodies: polyclonal antibody to mouse IgE (Fc specific)-peroxidase (HRP) (OriGene, Rockville, MD, USA; dilution 1:6000, 1.5 h at room temperature). The colorimetric reaction was developed by a solution containing 30 mg of o-phenylenediamine (Sigma-Aldrich)/10 mL of PBS. Absorbance was measured at 450 nm in the EnSpire Manager microplate reader (Perkin Elmer, Waltham, MA, USA). Based on standard curves, the amounts of AGE10 in homogenates were calculated and then converted to 1 mL of serum. 

### 2.3. The Statistical Analysis 

A contractual line established at 1000 µg/mL of serum AGE10 classified patients as those with high (above 1000 µg/mL) and low (below 1000 µg/mL) AGE10 levels. The AGE10 concentrations (described as high or low) in three studied groups, representing non-stroke, TIA, and ischemic stroke subjects were compared in pairs by χ^2^ test. The odds ratio (OR) and 95% confidence interval [CI] were calculated for each pair of comparisons. The statistical relationship between the two variables was assessed by Spearman’s rank coefficient (r). The outcomes were regarded as statistically significant for *p* < 0.05. Statistical calculations were performed using STATISTICA 13.1 (StatSoft. Inc., Hamburg, Germany).

## 3. Results

The raw data are provided in the supplementary materials (Table S1).

**Figure 1:**
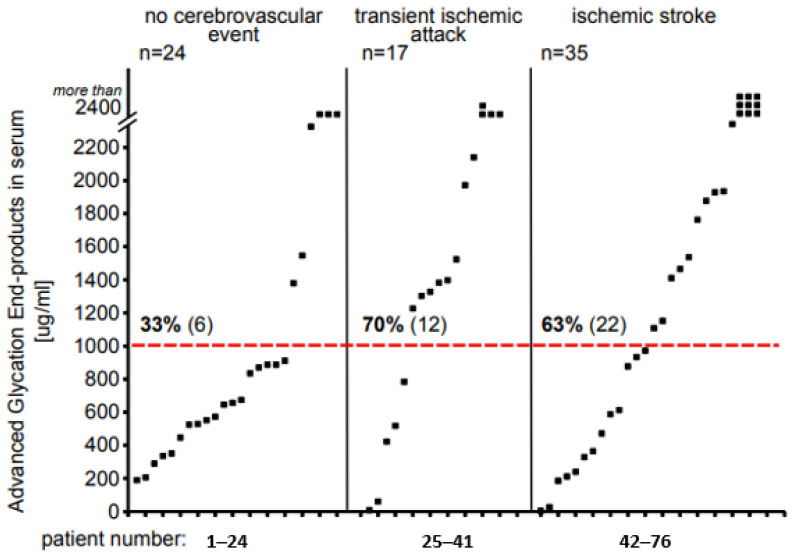
The scatter plot exhibiting the distribution of serum AGE10 concentrations (μg/mL) within groups of non-stroke, TIA, and ischemic stroke patients. The dotted red line divides patients into those with high (above 1000 μg/mL) and low (below 1000 μg/mL) AGE10 levels. χ^2^ test revealed that high AGE10 levels were found significantly more often in patients who experienced TIA and acute ischemic stroke (χ^2^ = 8.2, *p* = 0.004; χ^2^ = 8.0, *p* = 0.005, respectively) than in subjects with other (non-stroke) neurological disorders. The chance of ischemic incident was significantly risen in people with higher levels of AGE10s in blood (OR = 6.5, CI95%: 1.7–24.8; OR = 4.7, CI95%: 1.5–14.5 for TIA and stroke subjects, respectively).

A measurement threshold was established at 2400 µg/mL, on the basis of a standard curve. We observed vast differences in the AGE10 levels among the individual subjects, varying from undetectable levels up to concentrations higher than 2400 µg/mL. To facilitate the data interpretation, we set a borderline between low and high AGE10 values at 1000 µg/mL. Consequently, all the concentrations above 1000 µg/mL were considered high, and below this value as low. We found that in a group of participants without cerebrovascular event, only 33% of the people were characterized by high AGE10 concentrations (Figure 1). On the contrary, among the patients who experienced a cerebrovascular incident, both full-blown stroke and TIA, those with high AGE10 levels in the serum constituted 63% and 70% of the participants, respectively. An overrepresentation of high AGE10 concentrations among the patients with ischemic stroke and TIA in comparison to those who did not experience a cerebrovascular event was statistically significant (χ^2^ = 8.0, *p* = 0.005 and χ^2^ = 8.2, *p* = 0.004 for stroke and TIA subjects, respectively), and the risk of ischemic incident significantly rose in the people with higher levels of AGE10 in the blood (OR = 4.7, CI95%: 1.5–14.5 and OR = 6.5, CI95%: 1.7–24.8 for stroke and TIA subjects, respectively). Thus, an excess of AGE10-high subjects among stroke/TIA patients was striking when compared with people without stroke/TIA. 

**Figure 2:**
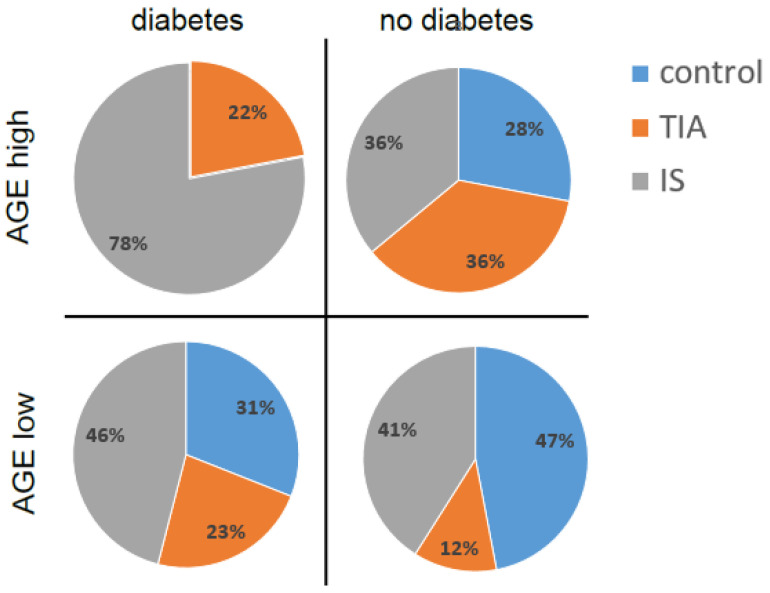
A division of patients with non-stroke neurological disorders (ctrl), TIA, and severe ischemic stroke (IS) among groups characterized by high and low AGE10 serum concentrations as well as presence or absence of diabetes. The cut-off point for defining low/high AGEs is 1000 µg/mL.

**Figure 3:**
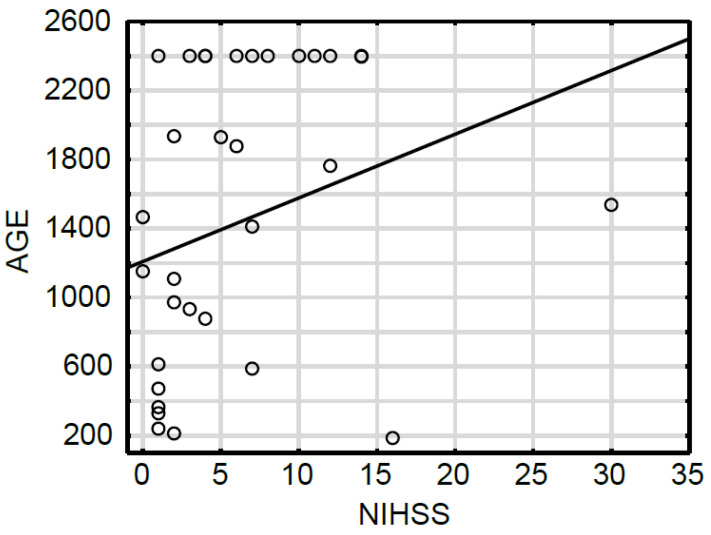
The Spearman rank correlation between AGE10 serum levels and neurological outcomes assessed by the NIHSS scale in patients with severe ischemic stroke (R = 0.35, *p* = 0.04) (the black line is the trend line).

All participants, both with and without cerebrovascular incident, were toted up and grouped into those with and without diabetes. Those groups were then subdivided into subjects presenting high and low AGE10 serum concentrations. It is striking that among the patients with diabetes exhibiting high levels of AGE10, 78% suffered from full-blown stroke (Figure 2). In addition, 22% experienced TIA, but no neurological patients without cerebrovascular event were identified in this group. The percent of AGE10 distribution was entirely different in a group of patients with high AGE10 concentrations but without diabetes. Thus, the subjects with severe cerebral ischemia and TIA accounted equally for 36% of the studied group. In addition, the non-diabetic people without cerebrovascular incident constituted 28% of the patients in this group. This shows clearly that participants with severe ischemic incident were strongly overrepresented among patients with diabetes and high AGE10 levels in comparison to people with high AGE10 content but without diabetes. On the contrary, such a relationship was not observed in patients presenting a low AGE10 content. Among the subjects with diabetes and low AGE10 levels, the group with severe stroke accounted for 46% of the studied people, which is only slightly more than in the non-diabetic group (41%). Interestingly, the patients who experienced TIA constituted 23% of the participants with diabetes and low AGE10 concentrations, and 12% (almost twice less) of the people without diabetes mellitus presenting a low AGE10 content. At the same time, subjects without cerebrovascular incident, suffering from diabetes, and presenting low AGE10 levels represented 31% of the total number of participants in this group. The patients without brain ischemia and exhibiting low AGE10 concentrations represented the largest group (47%) among the people without diabetes. All these results suggest that the coexistence of diabetes and high levels of AGE10s may highly predispose people to ischemic stroke. We also found a significant positive correlation (r = 0.40; *p* = 0.02) between diabetes and AGE10 concentrations in the serum of patients with stroke and a positive correlation between AGE10 concentrations and the National Institutes of Health Stroke Scale (NIHSS) (r = 0.35; *p* = 0.04) (Figure 3).

## 4. Discussion

Taking into account that AGE formation requires a sufficiently long period of time, it is rather unlikely that the analyzed AGE10 were created during (or soon after) the cerebrovascular incidents. Therefore, one can speculate that AGE10 may leak through the stroke-impaired blood–brain barrier (BBB), assuming that the central nervous system is enriched in the studied AGE10 in comparison with the blood. However, such speculations are not well grounded, since the overrepresentation of high levels of AGE10 was noted in people who experienced TIA, which does not lead to considerable BBB damage. Thus, high levels of AGE10 do not appear to be stroke-induced, but as the opposite: elevated AGE10 may substantially increase the risk of stroke. In our previous work, we revealed the lower level of circulating serum AGE10 in patients with Alzheimer’s disease in relation to healthy controls and the raised amounts of specific immune complexes in these patients [29]. 

Since AGE epitopes are formed by the glycation of proteins and lipids, the elevation of them is often associated with diabetes mellitus [7,31], the main feature of which is an increase in blood glucose content. This is an important observation because diabetes is one of the major risk factors for brain ischemia [32,33,34]. The results of this project, such as the correlation between AGE10 and diabetes, confirm the existence of the relationship. Moreover, AGE accumulation over the course of diabetes is considered one of the causes of diabetes-related vascular complications [35]. Following this line, poor diabetes control can enhance AGE10 production, which in turn may contribute to damage by AGE10 of cerebral vasculature, and as a consequence, lead to ischemic stroke. Diabetes is not only a risk factor for stroke occurrence. It is known that patients with diabetes who experienced ischemic stroke present with much worse outcomes than their non-diabetic counterparts. In our study, we also verified whether high levels of AGE10 influence the neurological outcome of patients after stroke incidence. Indeed, we noted the significant positive correlation between AGE10 concentrations and NIHSS, which indicated that high levels of AGE10 may worsen the condition of a patient with stroke. 

Especially noteworthy is the fact that AGE10 is a fairly recently discovered biochemical component of the body, so we do not yet have detailed knowledge of its structure, formation and metabolism. In recent years, there have been isolated reports on the diagnostic value of this antigen in various types of conditions [28,29], but the degree of our uncertainty about the mechanisms of the accumulation of this antigen is, we highlight, large. We do not know, for example, whether AGE10 in the blood can be of food origin or not. 

The main limitation of the present project is that the study sample size is not large (n = 76); thus, we present our direction for the future: to conduct this study on a much larger study group to confirm our inference on a large population of people with neurological diseases. Another limitation is the poor characterization of the samples, understood as the lack of additional medical information about a given patient. If the analyzed samples also had blood biochemistry parameters tested, such as glycated hemoglobin (HbA1c), fasting glucose, insulin, CRP, and others related to inflammation, the statistical analysis could be more thorough and could demonstrate the many detailed relationships between the analytes. 

The aforementioned HbA1c is currently actually the only routine blood biochemical parameter that refers to glycation and it was found to be an independent risk factor for the development of stroke in patients with and without diabetes [2,36]. Recently, glycated albumin has also been analyzed for its usefulness in strokes and TIA diagnosis [37]. We believe that AGE10 can enrich the diagnostic panel in estimating the risk of stroke and other vascular complications related to glycation. 

## 5. Conclusions

In conclusion, the AGE10 content can be considered as a novel risk factor for cerebral ischemic incidence and also could be principally important in patients with diabetes, indicating the subjects who present with a particularly high risk of cerebrovascular incident and could also be useful in the prediction of the neurological outcome of patients after stroke. Although it is still necessary to verify the sensitivity and accuracy and other parameters regarding the diagnostic value, which is planned to be performed on a large patient population, AGE10 currently seems to be a promising diagnostic tool, especially for neurological departments.

## Figures and Tables

**Table 1:** Baseline characteristic of the patients enrolled in the study.

	Control (n = 24)	Transient Ischemic Attack TIA (n = 17)	Ischemic Stroke IS (n = 35)
Females % (n)	54% (13)	76% (13)	66% (23)
Males % (n)	46% (11)	24% (4)	34% (12)
age median (Q1; Q3)	72 (53; 89)	78 (45; 91)	77 (48; 98)
concomitant conditions:			
diabetes %	17	30	51
hypertriglyceridemia %	30	37	34
hypercholesterolemia %	50	40	24
renal disease %	12	24	28

## Data Availability

Data supporting the reported results can be obtained from the corresponding author upon request.

## Supplementary Materials

The following supporting information can be downloaded at https://www.mdpi.com/article/10.3390/jcm13020443/s1, Table S1: Raw data: AGEs in patient serum.
